# The Sexually Dimorphic Adrenal Cortex: Implications for Adrenal Disease

**DOI:** 10.3390/ijms22094889

**Published:** 2021-05-05

**Authors:** Rodanthi Lyraki, Andreas Schedl

**Affiliations:** Université Côte d’Azur, Inserm, CNRS, Institut de Biologie Valrose, 06108 Nice, France

**Keywords:** adrenal cortex, sexual dimorphism, sex hormones, proliferation, adrenocortical carcinoma, Cushing’s syndrome, Addison’s disease, stem cells

## Abstract

Many adrenocortical diseases are more prevalent in women than in men, but the reasons underlying this sex bias are still unknown. Recent studies involving gonadectomy and sex hormone replacement experiments in mice have shed some light onto the molecular basis of sexual dimorphism in the adrenal cortex. Indeed, it has been shown that gonadal hormones influence many aspects of adrenal physiology, ranging from stem cell-dependent tissue turnover to steroidogenesis and X-zone dynamics. This article reviews current knowledge on adrenal cortex sexual dimorphism and the potential mechanisms underlying sex hormone influence of adrenal homeostasis. Both topics are expected to contribute to personalized and novel therapeutic approaches in the future.

## 1. Introduction

Many human diseases of nonreproductive organs display sex bias. While women are more susceptible to autoimmune diseases [1], men show higher incidence and worse prognosis in a wide range of cancers [2]. According to systematic phenotypic analysis of genetically modified mouse lines, sex is a major variable that determines a large proportion of mammalian phenotypic traits [3]. Many of the differences between males and females stem from the action of gonadal hormones, affecting stem cell activity, immune system activation, stromal compartments, and systemic metabolism [2,4,5]. In other cases, sexual dimorphism of mammalian traits has sex chromosome-related causes, such as the incomplete inactivation of X chromosome genes [6]. Although the importance of analyzing both sexes in biological studies has been overlooked in the past [7], the situation has been changing during the last ten years, and many major funding bodies now request that the impact of sex and gender are part of all relevant studies [8].

The adrenal cortex synthesizes steroid hormones that have a central role in coordinating the organism’s homeostasis, as part of the hypothalamic–pituitary–adrenal (HPA) axis and the renin–angiotensin–aldosterone system (RAAS). The outermost zone of steroidogenic cells in the adrenal cortex, the zona glomerulosa, synthesizes mineralocorticoids (most importantly, aldosterone), which regulate blood pressure and Na^+^–K^+^ balance. The second zone, the zona fasciculata, synthesizes glucocorticoids (predominantly, corticosterone in rodents and cortisol in humans), which regulate metabolism, immune system, and stress response. Finally, the innermost zona reticularis produces androgens (androstenedione and dehydroepiandrosterone—DHEA) in humans, but is absent in mice. Intense research has focused on the ability of the adrenal cortex to regenerate [9] and to regularly self-renew thanks to resident stem cell populations in the outer cortex, reviewed recently in [10,11]. Notably, the rate and capacity of self-renewal as well as several other physiological parameters of the adrenal cortex are markedly sexually dimorphic, owing mostly to gonadal steroid signaling (although genetic factors cannot be excluded). In this review, we summarize the current knowledge on the impact of gonadal hormones on the adrenal cortex and how it can affect susceptibility to adrenal disease.

## 2. Sex Bias in Adrenocortical Diseases

Many diseases of the adrenal cortex are more prevalent in women than in men. One of the most striking examples is Cushing’s syndrome, a set of pathologies that stem from the excess production of glucocorticoids (hypercortisolism). Hypercortisolism can have pituitary-related (Cushing’s disease) or extrapituitary causes (adrenocorticotropic hormone (ACTH)-independent Cushing’s syndrome), but is often linked to adrenal hormone-secreting tumors or nodular hyperplasia [12]. The overall female-to-male ratio in ACTH-dependent and independent Cushing’s is estimated at 3:1 [13,14], but sex bias appears more pronounced when examining specific causes of the disease. For example, a study of patients in Denmark estimates that Cushing’s syndrome due to benign adrenocortical adenomas is eight times more frequent in women than in men. Surprisingly, sex did not appear to influence mortality outcomes in this study [13]. Female bias is also observed in the incidence of other adrenocortical-specific etiologies of Cushing’s syndrome, such as bilateral macronodular adrenal hyperplasia (BMAH) (2–3 times more frequent in women) and primary pigmented nodular adrenocortical disease (PPNAD) [12]. Of note, while a 2-fold higher incidence in females is observed among PPNAD patients older than 12 years, this trend is reversed in children younger than 12 years, suggesting that the pubertal endocrine state largely determines the emergence of disparity between the sexes [12]. Further, in a large consortium of patients with Carney complex (a syndrome of multiple endocrine neoplasias), 73% of whom had inactivating mutations in *PRKAR1A*, PPNAD was more frequent and occurred earlier in women than men (71% vs. 29%) [15].

Adrenocortical tumors are, in general, more frequently diagnosed in women than in men independently of their histological subtype [16]. Among them, adrenocortical carcinoma (ACC) is a rare malignancy with poor prognosis that can cause Cushing’s syndrome [17]. The female-to-male ratio among patients diagnosed with ACC ranges from 1.5 to 2.5:1 [18,19,20,21,22], while women are associated with a higher prevalence of secreting tumors and a younger age at diagnosis [22]. Interestingly, a higher female-to-male ratio is observed both in adult-onset and in pediatric ACC (patients younger than 3 years). Nevertheless, puberty still has a strong effect on the amplitude of sexual dimorphism [23,24]. Thus, a combination of factors stemming from gonadal and chromosomal sex might influence the risk of developing a malignant adrenal tumor. The incidence of other types of adrenal tumors might also be influenced by sex. Adrenocortical incidentalomas, tumors that are diagnosed incidentally and are frequently hormonally silent, show a slightly higher prevalence in women. However, this could be attributed to an increased frequency of diagnostic procedures being performed on women [25,26]. In addition, somatic mutations in the gene *KCNJ5,* which encodes for a K^+^ channel, the most frequently identified mutations in aldosterone-producing adrenal adenomas (APAs) [27,28], are detected much more frequently in female compared with male patients (1.5–2.5:1) [29,30]. Interestingly, the adrenal cortex-specific loss of another K^+^ channel, TASK1, in mice, leads to a female-specific phenotype of primary aldosteronism and abnormal zonation. Pubertal androgens can rescue the phenotype in females [31]. As K^+^ channels regulate aldosterone secretion by the zona glomerulosa cells, it is possible that the female adrenal cortex is unable to compensate for the mutations by upregulating the expression of other K^+^ channels.

Of note, female bias is not only observed among adrenal hypersecretion diseases, but also among patients with Addison’s disease caused by adrenal hypofunction. In a study of 86 subjects with mostly autoimmune-related Addison’s disease, the male-to-female ratio was found to be 1:3.5 [32]. In another study of 94 patients with mainly autoimmune Addison’s, women predominate in the age group above 30 years [33].

## 3. Sexual Dimorphism in the Adrenal Cortex

### 3.1. Tissue Renewal and Homeostasis

According to studies on mouse and rat models, the mammalian adrenal cortex is a dynamic organ characterized by relatively high proliferation levels and regular cell renewal. Gender and age both affect the postnatal growth of the adrenal cortex. In pubertal and postpubertal mice of the C57BL6 strain, females consistently display higher adrenal weight and cortical volume than males. This difference has been attributed to a decrease in the volume of both adrenocortical zones after puberty in males, whereas in females the size of steroidogenic zones remains largely constant [34]. Notably, increased cell number, rather than cell size, is responsible for the sex-based difference in zona fasciculata volume [34]. Finally, the presence of a histologically defined area between the zona fasciculata and the medulla, termed the X-zone, is an important factor that differentiates male and female adrenal cortex in mice. The X-zone is a remnant of the fetal adrenal cortex that regresses after puberty in males but is retained in females until gestation [35].

In the mouse adrenal cortex, postnatal growth and renewal are attributed to several stem cell and progenitor populations in the capsule and zona glomerulosa and proliferating differentiated cells in the outer zona fasciculata [36,37,38,39,40]. An additional NESTIN^+^ cell population that can be found scattered throughout the cortex has also been reported to have progenitor activity, but their contribution to cell renewal under homeostatic conditions is likely to be limited [40]. In the rat adrenal cortex, an undifferentiated zone between the zona glomerulosa and zona fasciculata is thought to contain progenitor populations that fuel renewal of all steroidogenic zones [41], while cells expressing the pluripotency marker *Oct4* scattered throughout the cortex have been reported [42]. As lineage-tracing studies in rats have not been performed, the extent to which these cells contribute to tissue renewal is presently unknown.

Cell renewal is also subject to sexual dimorphism, and female mice display ~3 times higher adrenocortical turnover than males, owing partly to higher proliferative activity in all proliferating compartments [5]. Moreover, conditional lineage tracing of stem cell activity showed that the female adrenal cortex employs an additional progenitor population located within the capsule, characterized by the expression of *Gli1*, a marker of Sonic hedgehog (SHH) signaling. This population is mostly dormant in male adrenals, suppressed by circulating gonadal androgens [5]. Another study corroborates the suppressive effect of androgens on the adrenocortical renewal rate using a constitutive lineage tracing approach [43]. Extrapolating from these studies in mice, we can speculate that a higher rate of proliferation and stem cell recruitment may be the cause of female bias towards adrenocortical tumors in human patients.

### 3.2. Sexual Dimorphism in Mouse Models of Adrenal Disease

Interestingly, many mouse models of adrenal disease mimic the sex bias observed among human patients and can be useful to investigate its molecular causes. For instance, adrenal cortex-specific deletion of the *Prkar1a* gene, encoding for the regulatory subunit of the protein kinase A (PKA) complex, leads to pituitary-independent Cushing’s syndrome and hyperplasia in adult female mice [43,44]. These mutants display not only hypercortisolism but also an improper expansion of abnormal fetal cells that possess steroidogenic activity. In contrast, male mice show milder symptoms that appear later in age; however, gonadectomy can aggravate the phenotype in adult males, while androgen supplementation reverses endocrine hyperactivity [43,44]. Another example of female-predominant phenotype appears in the adrenal cortex-specific constitutive β-catenin activation model, which presents with ectopic zona glomerulosa formation, primary hyperaldosteronism, increased proliferation, and increased incidence of malignancy among aging mice. In contrast, male mutant mice consistently show slower phenotype progression compared with females and evade cancer risk [45].

Other genetic manipulations that affect the adrenal cortex physiology manifest themselves more strongly in male mice. For instance, steroidogenic cell-specific deletion of *Yap* and *Taz*, encoding for transcriptional co-regulators functioning downstream of the Hippo pathway, induces male-specific cortical atrophy progressively accompanied by hypertrophic eosinophilic nodules as observed in PPNAD. Presumably, exhaustion of the stem cell compartment is the cause of this atrophy [46]. In addition, deletion of *Ezh2*, encoding for a histone methyltransferase, in steroidogenic cells results in zona fasciculata-specific cortical atrophy and primary glucocorticoid insufficiency. In this case, improper maintenance of steroidogenic cell differentiation status is the probable reason for the atrophy [47]. While male mice recapitulate these phenotypes, they additionally present with aldosterone deficiency, and deregulation of different transcripts compared with females [47]. Thus, zona fasciculata identity and steroidogenic potential might be differentially regulated in the two sexes.

Based on the aforementioned studies, the general pattern that emerges is that female mice are more prone to diseases of adrenocortical hyperactivity and tumorigenesis, while male mice are more prone to adrenocortical atrophy. However, not all adrenocortical tumor models display female bias: in a model of metastatic ACC induced by the combination of *Trp53* deletion and β-catenin constitutive activation, no difference in the timing of tumor onset was observed between the sexes [48]. In another ACC model, due to *Igf2*/H19 loss of imprinting and *Apc* deletion, hyperplasia is observed first in males [49]. Moreover, there emerges a prominent role for circulating gonadal hormones in these differences. It is, however, apparent that mouse models of sexually dimorphic adrenal disease present with complex phenotypes, making it difficult to dissect the exact impact of gonadal hormones on adrenal physiology. Indeed, there is evidence that androgens not only affect cell proliferation and turnover, but also steroidogenesis and the persistence of fetal-like zones in the adrenal cortex. To make matters more complicated, it is still not clear which of these aspects are regulated as an effect of direct sex steroid signaling in the adrenal cells, and which of them are indirectly regulated via endocrine interactions (the HPA axis). In the next chapter, we will summarize studies that attempt to elucidate these questions.

## 4. Impact of Gonadal Steroid Signaling in the Adrenal Cortex

### 4.1. Expression of Sex Steroid-Related Genes in the Healthy Adrenal Cortex

Sexual dimorphism in organs is generally under control of sex hormones (androgens, oestrogens, and progestogens) that are produced by gonads. To elicit their function in a given tissue, steroids need to bind to their cognate receptors, which in turn activate or repress transcriptional targets. The cognate receptor for gonadal androgens, androgen receptor (AR) is detected at the transcriptional and the protein level in the adrenal cortex of rats and mice of both sexes [50,51,52]. Regarding the estrogen receptors that mediate their transcriptional effects, ERα (*Εsr1*) transcript and protein are detected in the rat adrenal cortex at much higher levels than that of ERβ (*Esr2*) [50,53,54]. On the contrary, studies on human adrenals have shown that ERβ is the predominant estrogen receptor in the cortex, during both puberty and adulthood, and that it is localized in all three zones of the adult cortex [55,56,57,58]. Whether this differential expression pattern points to different actions of estrogen signaling in humans and rodents is presently unknown. Importantly, ERβ in prepubertal and pubertal adrenal cortices is localized mainly in the zona reticularis and the fetal zone, suggesting a role in DHEA-S secretion during fetal life and adrenarche [55]. *GPER1*, the gene encoding for a transmembrane receptor associated with nongenomic, estrogen-dependent G protein signaling, is expressed in the rat and human adrenal (localized in the medulla and the zona glomerulosa in humans [50,55,56]. Finally, one study demonstrated high levels of progesterone receptor (PR) in the human adrenal cortex that was further increased in benign adenomas, and isolated PPNAD nodules [58].

Genes related to sex steroid metabolism are also expressed in the adrenal cortex. For example, three isoforms of steroid 5α-reductase (*Srd5a1*, *Srd5a2*, and *Srd5a3*), an enzyme catalyzing the conversion of testosterone to the physiologically highly active dihydrotestosterone (DHT), are detected in the rat adrenal cortex at the transcript level [50]. The gene encoding for aromatase (*Cyp19a1*), an enzyme involved in the transformation of androgens to estrogens, is also expressed in the rat adrenal cortex, albeit at very low levels compared with the reproductive organs [50,53]. CYP19A1 is localized in the zona glomerulosa and the medulla in young human adrenals, and its expression is increased during adrenarche [55]. Importantly, the presence of aromatase in these regions shows the possibility of local estrogen production and paracrine action.

Overall, these studies demonstrate that the molecular machinery required not only for reception and activation of gonadal steroid signaling but also for the local production (in the case of estrogen in human adrenals) and transformation to more potent forms (in the case of testosterone) is present in the mammalian adrenal cortex.

### 4.2. Gonadal Hormones and Adrenal Steroidogenesis

It has been well documented that premenopausal women have a lower risk of high blood pressure and cardiovascular disease compared with age-matched men (reviewed in [59]). This disparity has been attributed to the cardioprotective role of estrogens via complex effects on the renin–angiotensin–aldosterone system (RAAS) and the sympathetic nervous system. Among these effects, estrogens likely regulate the synthesis of aldosterone, one of the major effectors of the RAAS axis, by the adrenal zona glomerulosa. In particular, estradiol (E2) treatment of ovariectomized rats suppresses angiotensin II (AngII)-stimulated aldosterone secretion, in addition to decreasing the expression of AT1 receptor, the best-studied receptor of AngII in the adrenal cortex [54,60,61]. In addition, E2 treatment leads to increased expression of the AT2 receptor, an alternative binding site of AngII with less well characterized functions [61]. Caroccia et al. used an aldosterone-secreting adrenal cell line to show that E2 inhibits aldosterone synthesis when acting via ERβ. However, when this receptor is pharmacologically blocked, E2 instead stimulates aldosterone secretion via GPER1 [56]. This observation might be crucial in the case of APAs, where GPER1 expression is increased [56]. On the other hand, another study showed a stimulatory effect of DHT treatment on aldosterone secretion using another adrenal cancer cell line, notably via an AR-independent pathway, but no such effect of E2 [62]. Thus, male- and female-predominant sex hormones likely have opposite effects on aldosterone secretion, but these are context-specific and depend on which receptors are activated.

Rodent studies have shown that females generally display a more robust neuroendocrine response to stress, with higher circulating corticosterone and ACTH levels than those of males (reviewed in [63]). Sex hormones in rodents influence the HPA axis at many different levels, including the activation of gene expression at the hypothalamic paraventricular nucleus (PVN), the expression of ACTH precursor protein proopiomelanocortin (POMC) in the anterior pituitary, and the negative feedback by circulating glucocorticoids in these central organs. Therefore, it can be hard to dissect the effects of circulating gonadal hormones specifically on the adrenal cortex. In general, estrogens are thought to potentiate the HPA axis, while androgens inhibit its activation. Removal of most endogenous androgens by orchiectomy increases basal and stress-induced corticosterone and ACTH levels in the blood, while ovariectomy has the opposite effect [64]. Importantly, sex hormone replacement can reverse these effects [65].

As sex hormones affect the hypothalamus and pituitary, it has been difficult to determine whether they also act directly on the adrenal cortex. However, studies by Figueiredo et al. [66] in ovariectomized rats suggested that E2 has peripheral actions and is involved in augmenting adrenal sensitivity to ACTH. Moreover, earlier in vitro studies on primary adrenocortical cells from rats suggested that E2 directly augments basal corticosterone secretion, while treatment with high testosterone concentrations decreases ACTH-stimulated corticosterone secretion [67]. Curiously, orchiectomy in a nonlaboratory species (the sand rat *Psammomys obesus*) results in increased circulating ACTH and luteinizing hormone (LH), but lower circulating corticosterone, effects reversible by testosterone replacement [68]. In this case, an adrenal-specific effect of androgens (protection of zona fasciculata cells from apoptosis) might be involved in the observed drop in corticosterone production. Overall, these studies indicate that sex hormones affect glucocorticoid synthesis at both the central and peripheral (adrenal) levels. The observed effect of gonadectomy and hormone replacement can vary dramatically based on the experimental conditions and the type of stressor, and when these parameters differ, even opposite effects of the hormones can be observed [69].

Data on sexual dimorphism regarding the human HPA axis are much less consistent. While puberty-dependent differences in basal salivary cortisol levels between boys and girls have been reported, these can be attributed to differences in cortisol metabolism and elimination rate [70,71]. Testosterone replacement in men (previously under pharmacologically induced testosterone deficiency) leads to lower corticotropin-releasing hormone (CRH)-stimulated cortisol, suggesting a decreased adrenal sensitivity to ACTH [72]. On the other hand, a more recent study examined plasma cortisol and ACTH responses to psychosocial stress in men versus women (at the follicular phase of the menstrual cycle, when progesterone levels are similar to those of men) [73]. They found that men show greater cortisol and ACTH responses, although men with higher testosterone levels showed a lower stress response. These confusing results underline the complexity of hormonal responses and interorgan communication, and further studies will be required to better define the impact of sex hormones on the HPA axis and glucocorticoid production.

### 4.3. Effects of Abolishing Androgen Receptor Signaling in the Adrenal Cortex

Deleting the androgen receptor gene *Ar* in the rodent adrenal cortex is a useful way to investigate the direct effects of canonical androgen signaling in this tissue. Adrenals from male mice with global *Ar* deletion are hypertrophic and display lower apoptosis and higher proliferation than their wild-type counterparts. Moreover, *Ar* deletion results in higher plasma corticosterone and ACTH [74]. While these phenotypes corroborate previously mentioned studies on the suppressive effect of androgens on the HPA axis, they are probably linked to the receptor’s pituitary function. A later study analyzed an adrenal cortex-predominant deletion of *Ar* [51]. Compared to wild-type males, adrenals from male *Ar* knockout (KO) animals are hypertrophic, as was shown in the previous study, and there is a tendency for higher circulating corticosterone in ageing animals [51]. The observed hypertrophy is likely due to the abnormal retention of the X-zone [75], a transient remnant of the fetal cortex that regresses during puberty in males and gestation in females [35].

In addition to hypertrophy, *Ar* KO adrenal cortex displays other complex manifestations, including a reduction in apoptosis, zona fasciculata hypoplasia, and tissue degeneration during ageing [51]. Apoptosis is a vital part of tissue renewal in the adrenal cortex, which helps to clear out older cells that likely accumulate damaging by-products. This can explain how a reduction in apoptosis in the *Ar* KO model is associated with zona fasciculata thinning. Nevertheless, it should be noted that gonadectomy in male animals from a nonlaboratory species (the sand rat *Psammomys obesus*) leads to an increase in zona fasciculata apoptosis [68]. Therefore, the effects of AR signaling in the adrenal cortex of rodents can be complex and species-specific. Further analysis of *Ar* KO models will be required to determine the exact role of androgens in cell proliferation and tissue renewal in the adrenal cortex.

### 4.4. Role of Oestrogen Signaling in Telomere Maintenance in the Adrenal Cortex

We mentioned above that the female adrenal cortex is bigger and shows higher rates of proliferation and tissue turnover compared with male mice. More rigorous telomere maintenance could be one of the contributing factors to these sex differences. Telomere length, ensured by the enzyme telomerase, regulates the proliferative lifespan of a cell (reviewed in [76]). Estrogen deficiency, induced by a global deletion of aromatase, reduces telomerase activity specifically in the adrenal cortex of female mice, but not in other tissues [77]. Reduction of telomerase activity in this mouse model results in shorter telomeres, reduced cortical proliferation, and adrenal atrophy, which is reversible by estrogen replacement [77]. Human adrenals harbor very low levels of telomerase activity. Despite this, older men (>65 years old) have significantly shorter telomere lengths in the zona fasciculata compared with the corresponding female subjects [78]. Telomere attrition in ageing men might contribute to the reduction in adrenal weight that is observed in this age group [78]. Of note, zR displays the longest telomeres among the adrenocortical zones, and their length appears to increase rather than decrease with age [79]. Although these interesting studies were cross-sectional, in combination with the mouse data, they suggest an essential role of telomere maintenance in determining sex-dependent adrenal size and proliferation.

### 4.5. Sex Hormones in the Context of Adrenocortical Tumors

Studies on human adrenocortical cancer cells have indicated the importance of sex steroid signaling for ACC and the potential therapeutic potential of its manipulation. Indeed, treatment with DHT at physiological concentrations has a growth-suppressing effect on ACC cells, as well as on cells from nonfunctioning adenomas [80]. On the contrary, treating the same cell line with physiological concentrations of E2 increased cell proliferation, while estrogen receptor antagonists caused growth arrest or apoptosis [81]. Even though ERβ is the predominant estrogen receptor in the human adrenal cortex, the expression of ERα is increased in some cases of ACC compared with normal adrenal tissue [57]. A cross-talk of ERα and IGF-II pathways has been suggested to mediate estrogen’s tumor-promoting potential [82]. More importantly, administration of the ER antagonist tamoxifen to mice with subcutaneous xenografts of ACC cells leads to a reduction in tumor volume [82]. Increased aromatase expression has been noted in ACC tissue [57] and feminizing adrenocortical tumors [83], suggesting local production via androgen aromatization. Therefore, cancer cells in the cortex might rely on a local circuit of estrogen production exerting autocrine and paracrine actions for their uncontrolled growth.

## 5. Conclusions and Outlook

**Figure 1:**
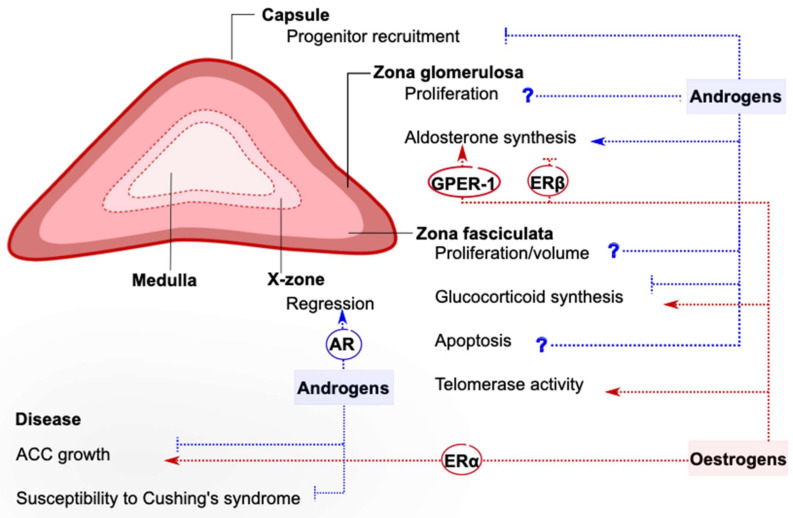
Reported effects of gonadal hormones on the mouse adrenal cortex. The sexual dimorphism characterizing many aspects of adrenal cortex physiology is attributed to circulating or locally produced androgens and estrogens, whose action can be inhibitory or stimulatory. Gonadal hormones can exert direct actions on the adrenal cortex, and sometimes the receptor responsible for initiating them has been recognized. In other cases, gonadal hormones act indirectly via inter-organ communication. Often, the effects of sex hormones are complex, and more research is needed to clarify their exact contribution (marked by a question mark). This figure summarizes knowledge derived from in vitro and rodent studies. AR: Androgen receptor, ERα: estrogen receptor α, ERβ: estrogen receptor β, GPER-1: G-protein-coupled estrogen receptor 1, ACC: adrenocortical carcinoma.

Sexual dimorphism in the adrenal cortex is mostly associated with endocrine interactions with gonadal hormones (summarized in Figure 1). Research has focused on androgens and estrogens, which appear to exert complex effects on steroidogenesis, stem cell activity, telomere maintenance, X-zone dynamics, and many other aspects of adrenal physiology. Gonadal hormones effects are clearer on ACC cells, when they appear to affect proliferation and growth of the malignant cells in opposite ways. However, crucial questions remain unanswered, including the nature of the observed effects (direct or indirect?) and the specific signaling pathways that enable them (transcriptional or nongenomic?). This knowledge is essential to promote personalized medicine and novel therapeutic approaches for patients with adrenocortical diseases in the future.

## Data Availability

Not applicable.
